# The impact of performance status and comorbidities on the short-term prognosis of very elderly patients admitted to the ICU

**DOI:** 10.1186/1471-2253-14-59

**Published:** 2014-07-22

**Authors:** Fernando G Zampieri, Fernando Colombari

**Affiliations:** 1 Unidade de Terapia Intensiva, Hospital Alemão Oswaldo Cruz, Rua João Julião, 133, São Paulo, São Paulo, Brazil; 2 Intensive Care Unit, Emergency Medicine Discipline, Hospital das Clínicas, University of São Paulo, São Paulo, Brazil

**Keywords:** Octogenarians, Critical care, Short-term prognosis, Performance status

## Abstract

**Background:**

Patients ≥80 years of age are increasingly being admitted to the intensive care unit (ICU). The impact of relevant variables, such comorbidities and performance status, on short-term outcomes in the very elderly is largely unknown. Few studies address the calibration of illness severity scores (SAPS3 score) within this population. We investigated the risk factors for hospital mortality in critically ill patients ≥80 years old, emphasizing performance status and comorbidities, and assessed the calibration of SAPS3 scores in this population.

**Methods:**

1129 very elderly patients admitted to a tertiary ICU in Brazil during a two-year period were retrospectively included in this study. Demographic features, reasons for admission, illness severity, comorbidities (including the Charlson Comorbidity Index) and a simplified performance status measurement were obtained. After univariate analysis, a multivariate model was created to evaluate the factors that were associated with hospital mortality. Alternatively, a conditional inference tree with recursive partitioning was constructed. Calibration of the SAPS3 scores and the multivariate model were evaluated using the Hosmer-Lemeshow test and a calibration plot. Discrimination was assessed using a receiver operating characteristics curve.

**Results:**

On multivariate analysis after stepwise regression, only the SAPS3 score (OR 1.08, 95% CI 1.06-1.10), Charlson Index (OR 1.16, 95% CI 1.07-1.27), performance status (OR 1.61, 95% CI 1.05-2.64 for partially dependent patients and OR 2.39, 95% CI 1.38-4.13 for fully dependent patients) and a non-full code status (OR 11.74, 95% CI 6.22-22.160) were associated with increased hospital mortality. Conditional inference tree showed that performance status and Charlson Index had the greatest influence on patients with less severe disease, whereas a non-full code status was prominent in patients with higher illness severity (SAPS3 score >61). The model obtained after logistic regression that included the before mentioned variables demonstrated better calibration and greater discrimination capability (AUC 0.86, 95% CI 0.83-0.89 versus AUC 0.81, 95% CI 0.78-0.84, respectively; p < 0.001) than the SAPS3 score alone.

**Conclusions:**

Performance status and comorbidities are important determinants of short-term outcome in critically ill elderly patients ≥80 years old. The addition of simple background information may increase the calibration of the SAPS3 score in this population.

## Background

Patients older than eighty years of age comprise a group of patients with increasing admittance rates to the intensive care unit (ICU) [1-4]. However, there is still reluctance to admit very elderly patients to the ICU, even when such admission is appropriate [5-7]. The short-term prognosis after intensive care is most likely the result of the interplay between illness severity, baseline patient characteristics (comorbidities, performance status [PS]) and the quality of care [2,8]. Although age appears to be an independent factor for mortality in the critically ill, it is uncertain whether specific age strata are associated with worse outcomes [2,9].

One of the most commonly used scoring systems is the SAPS3 scoring system [10]. Some limitations of SAPS 3 score in elderly patients should be mentioned. First, SAPS 3 does not separate between age groups in patients older than eighty years; i.e., there is no different punctuation for nonagenarians, which are grouped together with octogenarians [10]. Moreover, a SAPS3 score does not account for previous PS and for the global burden of comorbidities, which may be even more important in elderly patients [2]. Despite an overall good discrimination capability, there are concerns regarding the calibration of SAPS3 scores [11,12]. Some researchers reported that a trend towards an overestimation of mortality may be present [11,12]. Customization has frequently been applied in other studies to overcome these limitations, with promising results [12,13]. Regrettably, there are few data regarding the calibration and accuracy of SAPS3 scores for elderly patients because most reports use older scoring systems [1,3,4,8,14,15].

Therefore, we sought to explore the factors that are associated with hospital mortality in patients older than eighty years of age who were admitted to a tertiary ICU, with a special emphasis on the impact of comorbidities and PS. We hypothesized that PS and comorbidities would be associated with prognosis, independently of illness severity. We also sought to evaluate the calibration of the SAPS3 scoring system in this population and determine whether customization, through the addition of PS and comorbidities, would improve the calibration and prediction capability of the SAPS3 scoring system. Additionally, we assessed the performance of a new prognostic model in octogenarians that included only baseline comorbidities, performance status and admission type.

## Methods

This study was performed on patients from a tertiary 34-bed ICU in São Paulo, Brazil (Hospital Alemão Oswaldo Cruz). Demographical features as well as a simplified PS, comorbidities, reason for admission, need for organ support and outcomes (ICU and hospital deaths) are routinely collected within an automated database (Epimed Monitor®, Epimed, Rio de Janeiro, Brazil). The study was approved by the local ethics committee (*Instituto de Educação e Ciências*, *Hospital Alemão Oswaldo Cruz*), and due to its strictly observational and retrospective nature, informed consent was waived.

**Figure 1:**
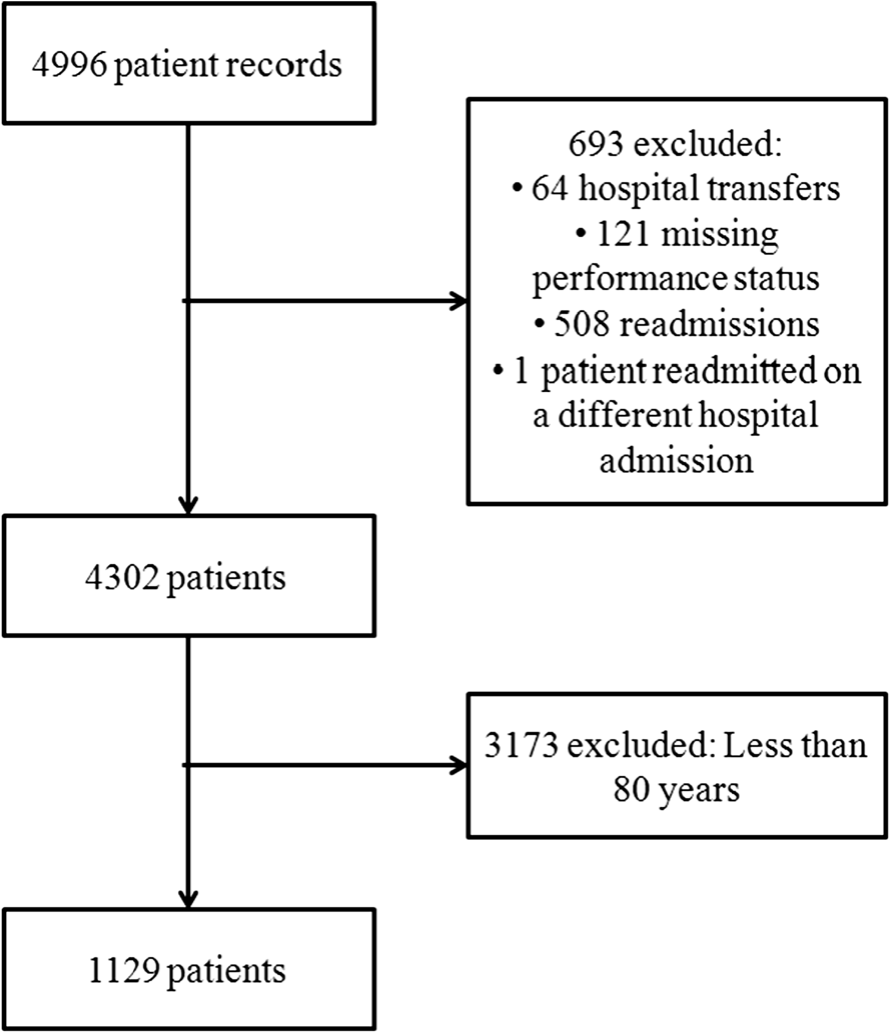
Study flowchart.

We included patients who were at least eighty years of age who were admitted to the ICU during a two-year period (from January 2012 until December 2013). Re-admissions were excluded from the analysis. Patients who were transferred to other hospitals were also excluded, as were patients whose PS data were absent from the database (Figure 1 – study flowchart). A simplified PS is routinely assessed at ICU admission, as previously described [16]. Briefly, a PS of 0 is used to define patients who are independent in terms of all basic daily activities. A score of 1 is attributed to patients who require assistance for at least one basic daily activity. A score of 2 is used for patients who are dependent for all basic daily activities. The Charlson Comorbidity Index was calculated as previously described, but without a correction for age [17]. A non-full code status was defined as any notation in the records that established a limitation for organ support, such as mechanical ventilation, renal replacement therapy, vasopressors or cardiopulmonary resuscitation. The use of organ support during the patient’s ICU stay was not included in the analysis because our main focus was the impact of the patient’s preexisting conditions and illness severity at admission.

Our primary endpoint was hospital mortality. Continuous variables were tested for normality using the Kolmogorov-Smirnov test. Parametric variables were compared between groups using a t-test, while non-parametric variables were compared using a Mann–Whitney test. Categorical variables were compared using a Chi-square test. All factors that were associated with mortality by univariate analysis with a p < 0.10 were included in a logistic regression analysis as shown on Table 1 (namely, we included age, SAPS 3 score, Charlson Commorbidity Index, body mass index, PS, admission type, specific clinical groups of the reason for admission, non-full code status and length of stay prior to ICU admission). The Charlson Comorbidity Index, but not the sole presence of each comorbidity, was added to the model because our main objective was to assess the impact of global measurements of health and performance rather than the impact of a specific comorbidity. A stepwise regression using a backward technique and the Akaike information criterion was performed. The final model consisted only of the variables significantly associated with hospital mortality after stepwise regression. Collinearity was assessed using variance inflation factor (VIF); a VIF value greater than 2.5 was arbitrarily defined as a marker of collinearity. When collinearity was present, the two variables with the highest VIF were selected, and the model was re-built with only one of the two variables at a time; the variable that was associated with the model with the greatest pseudo-R^2^ was retained. A 10,000-replication bootstrap of the model was then performed on the sample, and 95% bootstrap confidence intervals were collected. The discrimination of the final model was assessed using the area under curve (AUC) of the receiver operating characteristic (ROC) curve. Discrimination was also assessed using Somers’ D_*xy*_ rank correlation, and determination was evaluated through pseudo-R^2^. Calibration was assessed through Hosmer-Lemeshow test, with visual analysis of the calibration curve after 10,000 bootstrap replications with mean absolute error measurement. We also collected the same information regarding accuracy and prediction capability for isolated SAPS3 scores for a given sample. DeLong’s test was used for comparison between the AUCs of the ROC curves [18].

**Table 1:** Characteristics of the global population and comparison between survivors and non-survivors

**Variable**	**All patients (n = 1129)**	**Survivors (n = 915)**	**Non-survivors (n = 214)**	p*
**Age, median [IQ]** †	85 [82,88]	85 [82,88]	87 [84,90]	<0.001
**Sex, male (%)**	517 (45)	413 (45)	104 (48)	0.401
**SAPS 3, median [IQ]** †	54 [44–62]	51 [41,58]	67 [58,78]	<0.001
**Charlson Commorbidity Index, n [IQ]** †	2 [1–3]	1 [0,3]	3 [1,5]	<0.001
**Body mass index, kg/m**^**4**^**, median [IQ]** †	24.8 [22,28.1]	24.9 [22.2,28,3]	23.8 [20.6,27.4]	0.001
**Performance status†**				<0.001
0	400 (35)	363 (40)	37 (17)	
1	552 (49)	449 (49)	103 (48)	
2	177 (16)	103 (11)	74 (35)	
**Admission type, n (%)†**				<0.001
Medical	772 (68)	589 (64)	183 (85)	
Elective surgery	318 (29)	298 (32)	20 (10)	
Emergency Surgery	39 (3)	28 (4)	11 (5)	
**Specific reasons for admission**				
Sepsis†, n (%)	258 (23)	174 (19)	84 (39)	<0.001
Cardiovascular† n (%)	185 (16)	163 (17)	22 (10)	0.009
Respiratory n (%)	80 (7)	62 (6)	18 (7)	0.489
Neurologic n (%)	82 (7)	70 (7)	12 (6)	0.373
Renal† n (%)	28 (2)	17 (2)	11 (5)	0.011
**Non-full code status**†	80 (7)	15 (2)	65 (30)	<0.001
**LOS before ICU stay, days, median [IQ]** †	1 [0,1]	0 [0,1]	1 [0,3]	0.025

*p value between survivors and non-survivors. † variable added to the logistic regression.

In an alternative analysis, we applied automated conditional inference tree analysis (R package *party*, version 1.0-13) to understand the interplay between the measured variables and hospital mortality. Variables that were associated with outcome by univariate analysis were included in the conditional inference tree. Briefly, the conditional inference tree was built using recursive partitioning, where variables with the strongest association with the response were included in a decision tree on a stepwise basis. Cutoffs for dichotomization were automatically selected. The recursive partitioning was also adjusted using Monte Carlo simulation (10,000 times). The minimum criterion for node split was defined as 0.05, and the minimum bucket was defined as forty patients. The results are displayed as a single conditional inference tree.

A new simplified score was also created to assess how comorbidities and PS would behave in a model without SAPS 3 score to predict hospital mortality. In this score we did not include Charlson, but only the presence of comorbidities that were associated with outcome on univariate analysis (Additional file 1: Table S2). Length of stay before ICU was categorized in less or equal than one day or more than one day. The one day cutoff for length of stay before ICU admission was selected based on ROC curve inspection and Youden index calculation for the greatest discriminative capacity of previous length of stay regarding hospital outcome. The other variables included were the same of the main model displayed above, and statistical analysis was conduced the same way. In order to obtain a “user-friendly” model, we gathered the beta coefficients of all variables and divided then by the smallest beta coefficient obtained. The number of points attributed to each variable was the rounded value of the ratio between the variable’s beta coefficient on the logistic regression and the smallest beta coefficient obtained. For example, if the smallest coefficient obtained in the regression was 0.5 and a given variable had a beta coefficient of 1.08, this variable would be attributed +2 points (since 1.08/0.5 ≅ 2). Values were rounded to the closest values at 0.5 intervals. The same process was applied to all variables on this model. The score was named Elders Performance and Comorbidity Prognostic Score (EPCP Score). Its accuracy and calibration were assessed in the same way described above for the SAPS 3 score and the final model.

Finally, due to eventual bias that would result from from including patients with a non-full code status, a sensitivity analysis excluding patients that had any life support limitation during ICU stay was planned.

All analyses and graphics were performed using R project version 3.0.2 (http://www.r-project.org) with R Studio (version 0.97.551) and the following packages: *party*, *rms*, *car*, *pROC* and *OptimalCutpoints*.

## Results

A total of 1129 patients were included in this study (Figure 1), which accounted for 26% of all patients admitted to the ICU during the study period. Data for all patients and stratified according to hospital survival is shown on Table 1. Detailed information regarding comorbidities, reasons for admission and outcomes for the whole population are shown in the (Additional file 1: Table S1). The majority of the patients were non-surgical (772 patients, 68%), and sepsis was the most frequent clinical reason for admission (258 patients, 23%). The median Charlson Comorbidity Index was 2 (interquartile 1–3; range 0–12). Components of Charlson Comorbidity Index independently associated with mortality on univariate analysis are shown on (Additional file 1: Table S2). The most common support that was received was respiratory (15% used non-invasive ventilation, and 17% required mechanical ventilation). Vasopressors were used in 9% of patients, and renal replacement therapy was used in only 5% of all patients (Additional file 1: Table S1).

The non-survivors were older, were more severely ill, had more comorbidities (as assessed by the Charlson Comorbidity Index) and had a worse PS (Table 1). The presence of a non-full code status was also more common in the non-survivors. Patients who were admitted because of sepsis and renal complications had an increased mortality rate, whereas patients who were admitted for cardiovascular concerns tended to have a lower mortality rate. Non-surgical patients in general had a higher mortality rate. The LOS before ICU admission was higher for the non-survivors. After stepwise regression, only SAPS3 scores, Charlson Comorbidity Index scores, worse PS and a non-full code status were associated with hospital mortality (Table 2); these variables were used for the final model. The only variable that was present in the model after stepwise regression with a non-significant p-value and that was, therefore, not present in the final model was sepsis, with a p value of 0.07 and high collinearity with SAPS 3. Bootstrap analysis showed 95% confidence intervals that were similar to the original logistic regression, with a low boot bias, suggesting that there is a low probability of overfitting in this analysis.

**Table 2:** Results of final model and 10,000 replications bootstrap

**Variable**	**Odds ratio**	**95% CI**	**Bootstrap bias**	**Bootstrap 95% CI**	**P**
**SAPS 3, per point increase**	1.08	1.06-1.10	<0.001	1.061-1.095	<0.001
**Charlson commorbidity index, per point increase**	1.16	1.07-1.27	0.001	1.070-1.271	0.001
**Performance status**					
0	Ref	Ref	Ref	Ref	-
1	1.61	1.05-2.64	0.003	1.072-2.657	0.033
2	2.39	1.38-4.13	0.008	1.355-4.264	<0.001
**Non-full code status**	11.74	6.22-22.16	0.04	5.783-24.057	<0.001

**Figure 2:**
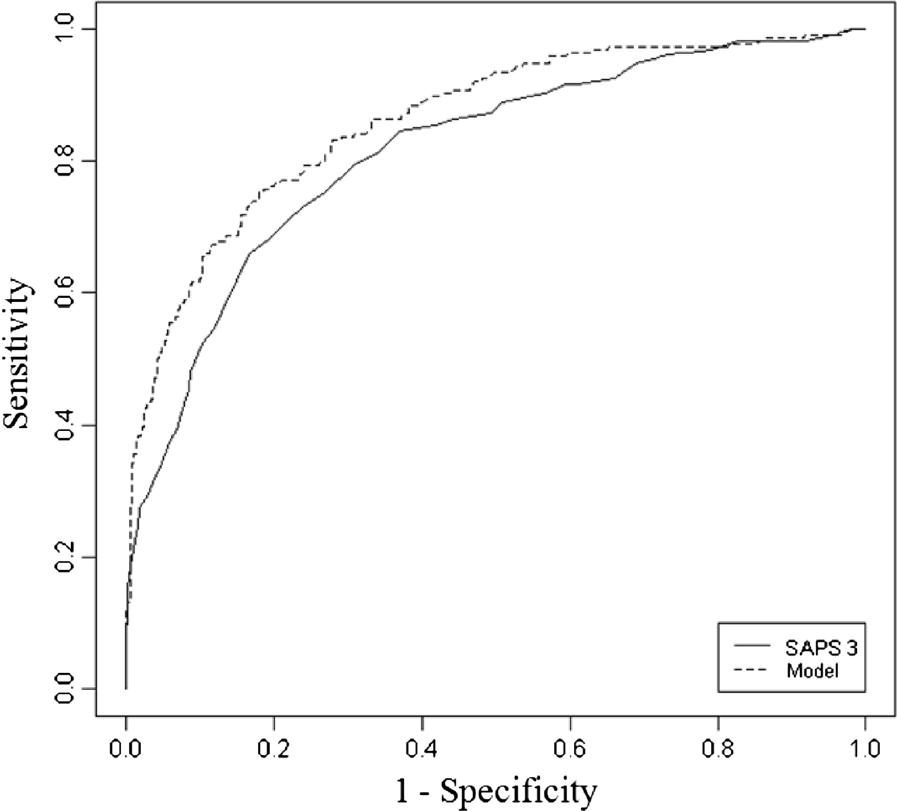
**ROC curve for SAPS 3 and created model on the studied population.** AUC for SAPS 3 = 0.81 (95% CI: 0.78-0.84). AUC for model = 0.86 (95% CI: 0.83-0.89). Curves are statistically different (DeLong’s test p < 0.001).

**Figure 3:**
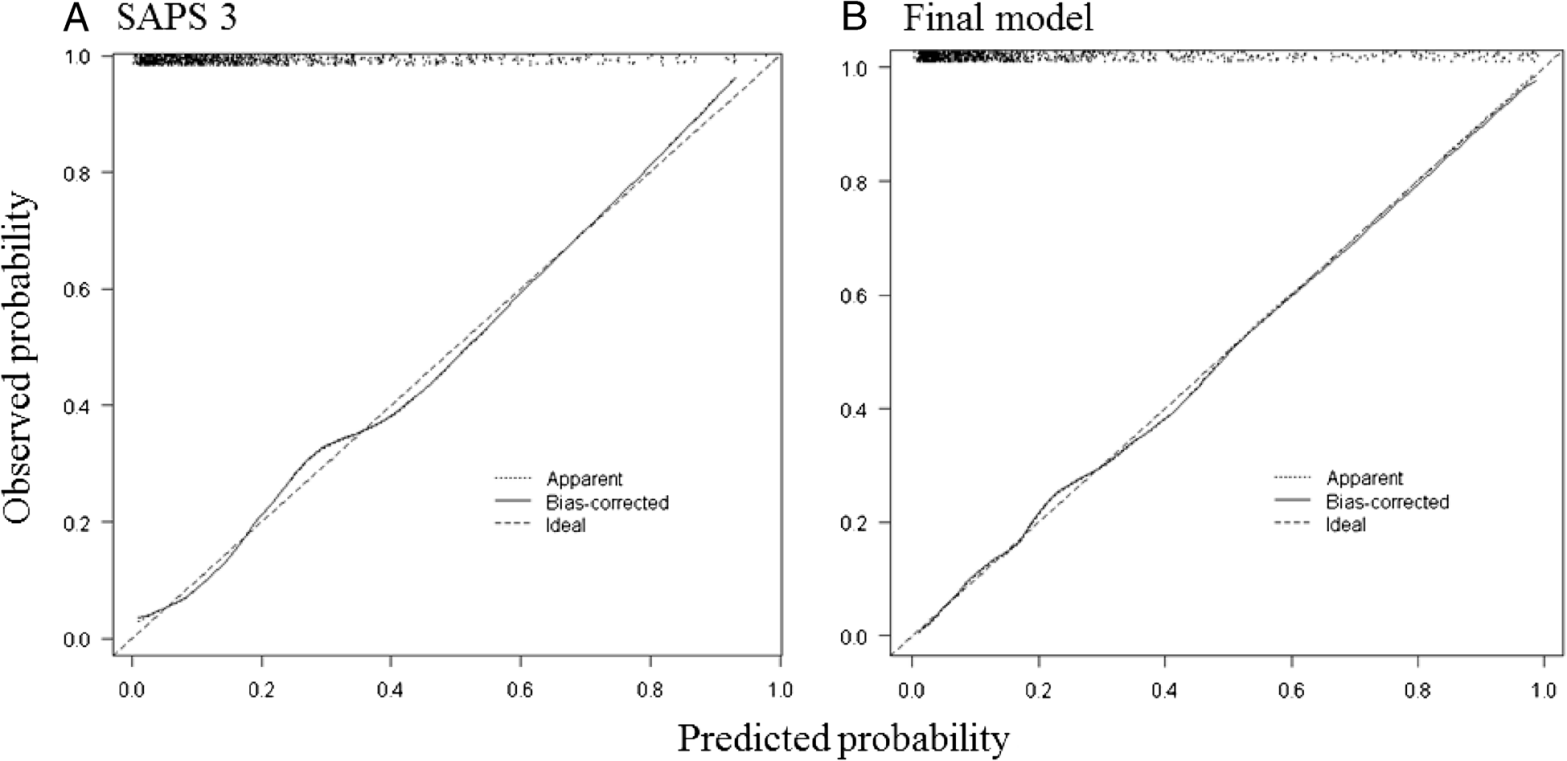
**Calibration plot after 10,000 bootstrap replications for predicted versus observed probability of hospital mortality of SAPS 3 (panel A) and the final model (panel B).** Note that SAPS 3 shows systematic errors around 0.3 and 0.5 of predicted probability (underestimation and overestimation, respectively) and systematic errors on extreme probabilities that era reduced in the model.

**Figure 4:**
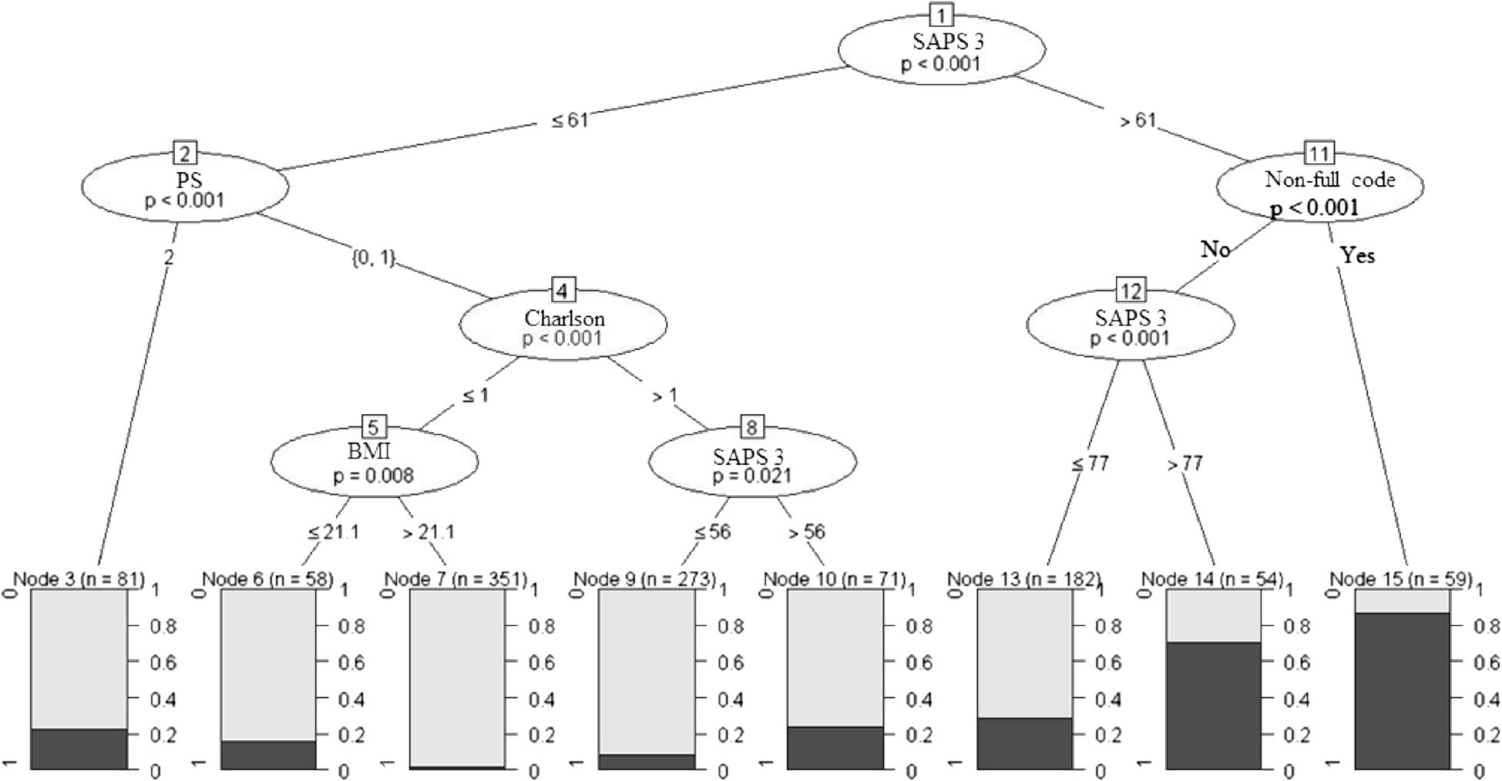
**Conditional inference tree using recursive partitioning results for hospital mortality.** Dark grey bars mark percentage of patients that died during hospital stay. p values for each node are shown inside the ellipsis.

The final model created had an AUC superior to the AUC of the SAPS3 scores alone (0.86, 95% CI 0.83-0.89 versus 0.81, 95% CI 0.78-0.84, respectively; p < 0.001; Figure 2). The discrimination and determination indices were higher for our model than for SAPS3 alone (Table 3). Both models were calibrated according to the Hosmer-Lemeshow test (model p = 0.71, SAPS3 p = 0.56); however, the calibration plot showed a better calibration and fewer systematic errors in the model compared with SAPS3 scores (Figure 3, panels A and B). In essence, SAPS3 showed systematic errors at approximately 0.3 and 0.5 (underestimation and overestimation, respectively, panel A in Figure 3), which were reduced by the model. The model abolished the systematic errors that were present in probabilities close to 0.30 and reduced the underestimation by approximately 0.5. Calibration on extreme values of probability was also improved by the model (Figure 3). Therefore, the final model had both better discrimination and calibration than SAPS 3 alone in our sample (Table 3 and Figures 2 and 3).The results of the conditional inference tree after recursive partitioning are shown in Figure 4. Variables that were selected by recursive partitioning were the same that were selected after logistic regression except for body mass index, which was selected by recursive partitioning and not by logistic regression (Figure 4). The most important variable that was associated with response was the SAPS3 score (first node). PS and Charlson Comorbidity Index were relevant to the outcome only in patients with lower illness severity (SAPS3 score ≤61, left branch of the tree). Specifically, only a PS of 2 was associated with poorer outcome on this branch. The Charlson Comorbidity index was an important discriminator between survivors and non-survivors in patients in the lower SAPS3 branch (≤61 points) and with a PS lower than 2. A low (≤21.1 kg/m2) body mass index was associated with mortality in patients with low Charlson Comorbidity Index (node 5). A non-full code status exerted an important discrimination on patients with higher SAPS3 scores (>61 points).

**Table 3:** Comparison of determination, discrimination and calibration indexes of SAPS 3 and created model to predict hospital mortality

**Variable**	**SAPS 3**	**Model**
**Pseudo R**^**2**^	0.32	0.43
Pseudo R^2^ after Bootstrap	0.32	0.42
**C statistic**	0.81	0.86
C-statistic after Bootstrap	0.81	0.85
**D**_***xy***_	0.62	0.71
D_*xy*_ after bootstrap	0.62	0.71
**Mean absolute error after bootstrap**	0.013	0.008

Results of the logistic regression used for creation of the EPCP Score are shown on Additional file 1: Table S3. The EPCP score, including beta values and corresponding points for calculation are shown on Additional file 1. In brief, EPCP punctuated for PS, admission type, cardiovascular reason for admission, chronic kidney disease, heart failure, metastatic tumor, hematological malignancy, LOS > 1 day and non-full code status The EPCP score ranges from 0–15. The score presented good accuracy (AUC 0.82; 95% CI 0.79-0.85 – Additional file 1: Figure S1), which was similar to SAPS 3 score (p = 0.518) and lower that the AUC of the final model (p = 0.001). The model was calibrated according to Hosmer-Lemeshow test (p = 0.804) but when the calibration plot was inspected, EPCP had more systematic errors than SAPS 3 score and the final model, especially a tendency to underestimate mortality on middle range probabilities and overestimation on higher death probabilities (Additional file 1: Figure S2).

Due to eventual bias that would arise from including patients with a non-full code status, we performed a sensitivity analysis excluding patients that had any life support limitation during ICU stay. 1049 (149 non-survivors) were included on this analysis. Results are shown on the (Additional file 1: Table S4). This analysis yielded similar results, except for PS of 1 which was no longer statistically associated with worst outcome, although it almost reached significance (p = 0.052; OR 1.63, 95% CI 0.99-2.69).

## Discussion

In a retrospective evaluation of a large number of critically ill patients above the age of eighty who were admitted to the ICU, only SAPS3 score, Charlson Comorbidity Index, PS and a non-full code status were independently associated with increased hospital mortality. Other variables that are usually associated with worse outcome, such as increased age and admission type, were not statistically associated with mortality. The model that incorporates the aforementioned variables improved the discriminative capability and calibration of the SAPS3 score. We complemented the traditional logistic regression with a recursive partitioning technique, thereby allowing us to understand the impact and interplay of specific variables on outcome. Using this analysis, we have shown that the effects of comorbidities (as assessed by the Charlson Comorbidity Index) and PS were more pronounced in patients who were less severely ill, whereas a non-full status was associated with a higher mortality mostly in patients who were more severely ill.

The prognoses of the critically ill elderly patients who are admitted to the ICU have recently been the subject of several analyses, and the association between illness severity and prognosis has been consistently reported. For example, Somme *et al*. concluded that illness severity, as assessed by APACHE II score, was the only predictor of short-term (hospital) mortality in elderly patients, while age and limitation of daily activities were the only predictors of long-term mortality [9]. Similar results were obtained by Boumendil *et al*., Vosylius *et al*. and Rooij *et al*. [2,4,8,14] who reported that short-term outcome was primarily dependent on the severity of the illness. Our analysis is in agreement with these findings. Regarding the impact of PS on outcome, the association is less clear. Other studies including different populations of critically ill patients highlighted the importance of PS or other measurements of poor performance (such as frailty) on outcome [19-21]. This association is especially true in oncologic patients [19,20]. In elders, Boumendil *et al*. found that performance status was associated only with long-term prognosis, while Roch *et al*. reported no clear association between PS and long-term outcome [3]. Bo *et al*. suggested that lack of independence was associated with worst hospital outcome, but the sample evaluated was relatively small and included patients over 65 years [22]. The reasons for the discrepancies in the literature may include not only differences in sample size and statistical analysis but also regional preferences regarding end-of-life policies and cultural heritage. Also, many studies included patients over 65 years and not only very elderly patients [4,22]. Using a large sample of very elderly patients, we were also able to show that PS and comorbidities may be important even for a short-term outcome such as hospital mortality.

Our study also suggests that the SAPS3 score has a good discrimination capability in critically ill elderly patients. The discrimination and calibration of SAPS3 has been the subject of previously reported analyses, but no studies have focused on a large population of critically ill elderly patients. SAPS3 is associated with poor calibration but has good discrimination capability [11,23]. The current analysis suggests that SAPS3 retains its discrimination capability even in very elderly patients. With regard to calibration, previous analyses usually relied on the Hosmer-Lemeshow test to assess calibration; despite its utility, this test is highly sensitive and usually displays better values in smaller series [24]. Other measurements of calibration, including a calibration plot after bootstrap, may provide a better understanding of calibration across different probabilities. Within a large unselected population of critically ill individuals, using a calibration plot, Nassar *et al*. demonstrated that SAPS3 tends to underestimate mortality on lower probabilities and overestimate mortality on larger probabilities; however, the analysis was limited due to the lower overall probability of death in this population [11]. We applied a slightly different approach because we also performed a bootstrap analysis of the calibration plot, but our results remain in agreement. We noticed systematic errors at approximately 0.3 (underestimation), 0.5 (overestimation) and on extreme probabilities (close to 0 and 1). Interestingly, the final model that we created (which included PS, Charlson Comorbidity Index and non-full code status) showed fewer systematic errors and excellent calibration even on extreme probabilities (Figure 3B). Therefore, we have shown that the calibration of severity scores in the elderly may be improved by the addition of simple background information regarding overall performance status, comorbidities and treatment limits.

We used a conditional inference tree analysis to better understand the interplay between variables and outcome. A similar approach was previously used by de Rooij *et al*. [14], but the authors focused on physiological variables and on the need for organ support, whereas we were more interested in overall global measurements of health and the burden of comorbidities. Recursive partitioning selected variables in a similar way as logistic regression. As shown in Figure 4, PS and the Charlson Comorbidity Index were discriminative only for patients with lower SAPS3 scores (<61). For patients in the left branch (SAPS3 ≤ 61), the second most important prognostic discriminator was a PS of 2, and a Charlson Index greater than 1 was discriminative in patients with a PS of 0 or 1. Low body mass index was associated with higher mortality in less severe patients, with PS < 2 and low Charlson Comorbidity Index. This association was not clear on the logistic regression and may be reflection of the impact of frailty on prognosis [21]. In contrast, a non-full code status was important on the right side of the tree (SAPS3 > 61 points). This finding was not unexpected because it is reasonable to conclude that very severely ill patients with life support limitations will have worse outcomes. Recursive partitioning was unable to show any impact of comorbidities and PS on the more severely ill patients. The results of the recursive partitioning suggest that the reduced underestimation of mortality of the lower death probabilities on the calibration plot of the model may be the result of the addition of PS and the Charlson Index, whereas the reduced underestimation of higher probabilities was most likely the consequence of the addition of a non-full code status to the model.

Additionally, we proposed a simple score to predict hospital outcome after ICU admission in octogenarians (EPCP Score) that did not include SAPS 3 score. Interestingly, this score had a performance that was similar to SAPS 3 score in terms of accuracy but that was inferior to the final model (that included SAPS 3, Charlson Charlson Comorbidity Index, PS and Non-full code status). EPCP Score displayed similar AUC and calibration to other prognostic scores in elderly patients [25]. This suggests that the interplay between the patient’s previous history and its performance status with the acute illness severity is probably more relevant than either illness severity alone or patient’s previous history. Validation of this score should be performed in adequate prospective studies.

In summary, we present evidence that the clinical background of very elderly patients is an important determinant of short-term prognostic. The two analyses presented are complementary: logistic regression highlights that PS, Charlson Comorbidity Index and non-full code are independently associated with hospital outcome and may improve SAPS 3 calibration, while recursive partitioning helps us understand the interplay between them and visualize on which particular type of patient each variable may be more significant. Recursive partitioning therefore provided clues to why PS, Charlson Comorbidity Index and Non-full code status improved calibration of SAPS 3 score.

There are, however, several limitations to our analysis. First, this study is a unicentric retrospective analysis and is therefore subject to local bias. Second, we measured only short-term outcomes and not long-term prognoses. Other outcomes, such as one- or five-year outcomes, may be more relevant to clinical practice [26]. Third, we were unable to measure PS after critical illness. Fourth, there may be a bias involved in the association between a non-full code status and mortality, since we were unable to control if patients received limitation in aggressive care only after there was a clear sign that they would not survive; nevertheless, even when non-full code patients were excluded from the analysis, the association between PS and Charlson with outcome was kept. Finally, most customizations of prognostic scores validated on a single sample display better calibration and discrimination capabilities than general prognostic scores that were developed on larger, different samples, since they tend to reflect the sample in which they were built. The most appropriate way to solve this bias would be through the validation of our model on a different, third sample unrelated to ours. We tried to minimize this bias by using a robust statistical analysis with bootstrapping and Monte Carlo replications in order to discard the presence of model overfitting; nevertheless, an independent validation of our results on other settings should be performed.

## Conclusions

A worse performance status, burden of comorbidities and a non-full code status were associated with worse outcome in critically ill patients over the age of 80. Comorbidities and performance status appeared to be more relevant for the patients who were less severely ill. The addition of this information to the SAPS3 score improved calibration and reduced systematic errors of the score.

## Abbreviations

SAPS 3: Simplified Acute Physiology Score 3; PS: Performance Status.

## Competing interests

The authors declare that they have no competing interests.

## Authors’ contributions

FGZ: Designed the study and collected data, performed statistical analysis, wrote the manuscript. FC: Helped on study design and data collection, revised manuscript. Both authors read and approved the final version of the manuscript.

## Pre-publication history

The pre-publication history for this paper can be accessed here:

http://www.biomedcentral.com/1471-2253/14/59/prepub

## Supplementary Material

Additional file 1: Table S1 Additional information regarding reason for admission, comorbidities and use of organ support during ICU stay of the whole patient sample. **Table S2.** Univariate analysis for the association between comorbidities and hospital mortality. **Table S3.** Results of the new simplified model and points attributed to each variable to the EPCP Score. **Figure S1.** ROC Curve for EPCP. AUC 0.82; 95% CI 0.79-0.85. **Figure S2.** Calibration plot for EPCP. **Table S4.** Results for multivariate analysis excluding patients with a non-full code status. Click here for file
