# Supplementary material for: The impact of performance status and comorbidities on the short-term prognosis of very elderly patients admitted to the ICU
Source: BMC Anesthesiol. 2014 Jul 22;14:59. doi: 10.1186/1471-2253-14-59 (PMC4112835; doi:10.1186/1471-2253-14-59)
Supplement: Additional file 1: Table S1 — Additional information regarding reason for admission, comorbidities and use of organ support during ICU stay of the whole patient sample. Table S2. Univariate analysis for the association between comorbidities and hospital mortality. Table S3. Results of the new simplified model and points attributed to each variable to the EPCP Score. Figure S1. ROC Curve for EPCP. AUC 0.82; 95% CI 0.79-0.85. Figure S2. Calibration plot for EPCP. Table S4. Results for multivariate analysis excluding patients with a non-full code status. [file 1471-2253-14-59-S1.docx]

***Additional File***

**Supplementary Table S1**: Additional information regarding reason for admission, comorbidities and use of organ support during ICU stay of the whole patient sample

| Reasons for medical admissions, n (%) |  |
| --- | --- |
| Sepsis | 258 (23) |
| Domiciliary Pneumonia | 152 (13) |
| Nosocomial pneumonia | 23 (2) |
| Urinary tract | 31 (3) |
| Bloodstream | 13 (<1) |
| Other | 39 (3) |
| Cardiovascular | 185 (16) |
| Uncompensated heart failure | 51 (4) |
| Supraventriclar arrhythmia | 51 (4) |
| NSTEMI/Unstable angina | 29 (3) |
| STEMI | 3 (<1) |
| Bradyarrhythmia/syncope | 23 (2) |
| Hypertensive emergency | 6 (<1) |
| Other | 22 (2) |
| Respiratory | 80 (7) |
| COPD | 25 (2) |
| Acute pulmonary edema | 13 (1) |
| Pulmonary embolism | 11 (1) |
| Asthma | 11 (1) |
| Other | 20 (2) |
| Neurologic | 82 (7) |
| Ischemic stroke | 34 (3) |
| Hemorrhagic stroke | 10 (<1) |
| Seizures | 17 (1) |
| Delirium | 9 (<1) |
| Other | 12 (1) |
| Renal | 28 (2) |
| Uncompensated chronic kidney  Failure | 19 (1) |
| Acute kidney injury | 9 (<1) |
| Pancreatitis | 22 (2) |
| Comorbidities, n(%) |  |
| Hypertension | 773 (68) |
| Diabetes | 352 (31) |
| With end-organ injury | 64 (5) |
| Heart failure | 189 (17) |
| NY Class 2 or 3 | 183 (17) |
| NY Class 4 | 6 (<1) |
| Previous myocardial infarction | 138 (12) |
| Stroke | 117 (11) |
| Dementia | 176 (16) |
| Chronic kidney disease | 241 (21) |
| On dialysis | 32 (2) |
| Liver failure | 6 (<1) |
| Child-Pugh C | 1 (<1) |
| Neoplasic disease | 235 (21) |
| Metastatic disease | 51 (4) |
| Hematologic disease | 34 (3) |
| COPD | 26 (2) |
| Need for organ support, n(%) |  |
| Mechanical ventilation | 192 (17) |
| Tracheostomy during ICU stay | 19 (1) |
| Duration of MV, days, median [IQ] | 2 [1-4] |
| Non-invasive ventilation | 176 (15) |
| Renal replacement therapy | 60 (5) |
| Vasopressors | 101 (9) |

***EPCP Score***

**Supplementary Table S2:** Univariate analysis for the association between comorbidities and hospital mortality

| Comorbidity | P value |
| --- | --- |
| Hypertension | 0.411 |
| Diabetes | 0.236 |
| With end-organ injury | 0.151 |
| Heart failure | <0.001 |
| Previous myocardial infarction | 0.700 |
| Stroke | 0.281 |
| Dementia | <0.001 |
| Chronic kidney disease | <0.001 |
| Liver failure | 0.154 |
| Neoplasic disease | <0.001 |
| Localized disease | 0.643 |
| Metastatic disease | <0.001 |
| Hematologic disease | <0.001 |
| COPD | 0.426 |

**Supplementary Table S3:** Results of the new simplified model and points attributed to each variable to the EPCP Score:

*The variables that were initially included on the logistic regression in order to built the model were age, body mass index, PS, admission type, admission due to sepsis, cardiovascular reason for admission, renal reason for admission, previous diagnosis of heart failure, chronic kidney disease, dementia, metastatic tumor, hematological malignancy and a non-full code status.*

*Of the initially included variables, the following variables were retained after stepwise regression and were included on the EPCP Score, PS, admission type, cardiovascular reason for admission, chronic kidney disease, heart failure, metastatic tumor, hematological malignancy, LOS > 1 day and non-full code status.*

| Variable | **β coefficient** | **Odds ratio** | **95% CI** | **P** | **Points for EPCP S** |
| --- | --- | --- | --- | --- | --- |
| Performance status |  |  |  |  |  |
| 0 | Ref | Ref | Ref | - | 0 |
| 1 | 0.59 | 1.81 | 1.16-2.84 | 0.008 | +1 |
| 2 | 1.28 | 3.63 | 2.13-6.17 | <0.001 | +2 |
| Admission type |  |  |  |  |  |
| Elective surgery | Ref | Ref | Ref | Ref | 0 |
| Emergency Surgery | 1.67 | 5.31 | 2.09-13.49 | <0.001 | +2.5 |
| Medical | 1.19 | 3.31 | 1.91-5.74 |  | +2 |
| Cardiovascular reason for admission | -0.81 | 0.44 | 0.25-0.77 | 0.004 | -1 |
| Chronic kidney disease | 0.69 | 2.00 | 1.34-3.00 | <0.001 | +1 |
| Heart failure | 0.48 | 1.63 | 1.04-2.53 | 0.030 | +1 |
| Metastatic tumor | 1.21 | 3.37 | 1.64-6.88 | <0.001 | +2 |
| Hematological malignancy | 1.24 | 3.48 | 1.59-7.63 | 0.001 | +2 |
| LOS > 1 day | 0.60 | 1.83 | 1.22-2.74 | 0.003 | +1 |
| Non-full code status | 2.75 | 15.69 | 8.45-29.14 | <0.001 | +4.5 |

**Supplementary Figure S1**: ROC Curve for EPCP. AUC 0.82; 95% CI 0.79-0.85


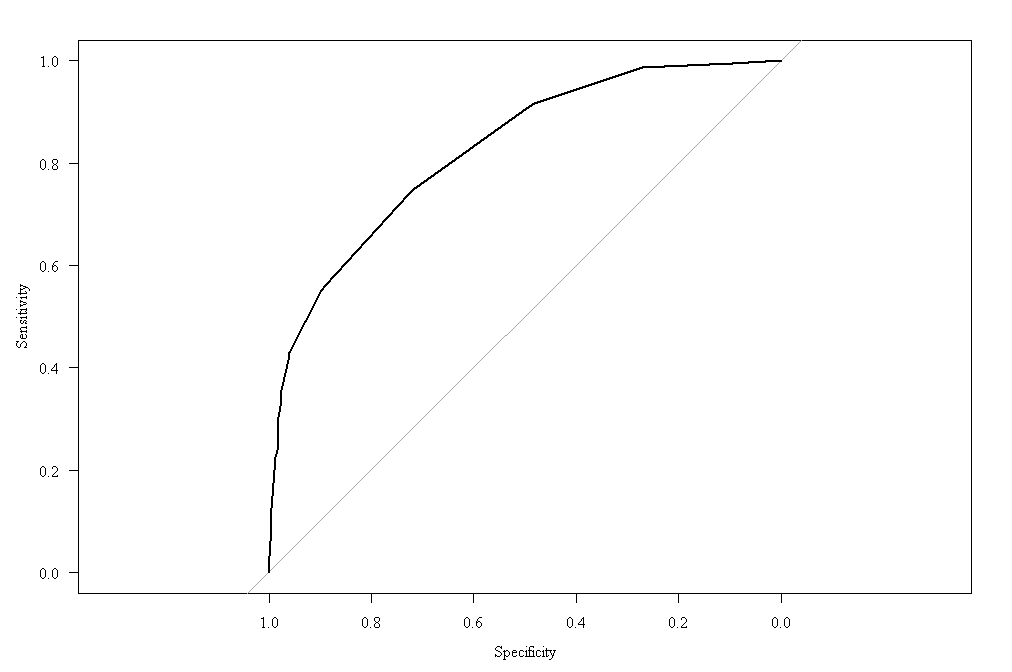


**Supplementary Figure 2:** Calibration plot for EPCP


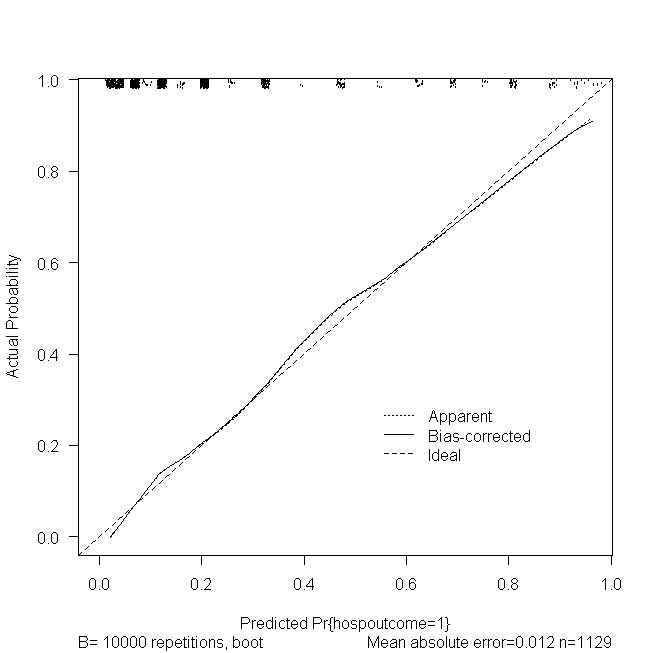


**Supplementary Table S4**: Results for multivariate analysis excluding patients with a non-full code status

| Variable | **Odds ratio** | **95% CI** | **P** |
| --- | --- | --- | --- |
| SAPS 3, per point increase | 1.07 | 1.06-1.09 | <0.001 |
| Charlson Commorbidity Index, per point increase | 1.17 | 1.07-1.28 | 0.001 |
| Performance status |  |  |  |
| 0 | Ref | Ref | - |
| 1 | 1.63 | 0.99-2.69 | 0.052 |
| 2 | 3.11 | 1.75-5.53 | <0.001 |
